# Allied health in residential aged care: Using routinely collected data to improve funding opportunities

**DOI:** 10.1111/ajag.13136

**Published:** 2022-09-07

**Authors:** Isabelle Meulenbroeks, Karla Seaman, Magdalena Z. Raban, Johanna Westbrook

**Affiliations:** ^1^ Australian Institute of Health Innovation Macquarie University Sydney New South Wales Australia

**Keywords:** Allied health occupations, Electronic health records, Homes for the aged

## Abstract

Consumers and providers have long been advocating for increased access to and delivery of allied health services in Australian residential aged care (RAC). There is significant evidence that allied health interventions are effective; however, there is limited evidence on the benefit of routine day‐to‐day allied health service delivery in RAC. This information is critical to effectively inform funders and policy advisors of the necessity of allied health in RAC. To improve arguments for future funding opportunities, providers, facilities and consumers need to partner together to use routinely collected, yet disparate, data, in electronic health and billing records, to improve data collection practices and evidence generation on allied health in aged care.

**Policy Impact**

The limited availability of data and evidence on allied health services in residential aged care facilities does allied health a disservice in current funding opportunities. In the long‐term national policy reform is required to address the allied health data drought and consistently generate evidence of allied health benefit.

**Practice Impact**

In the short term, to generate a stronger evidence base, provider, consumer and research partnerships should be created to leverage existing data on allied health in electronic health records.

## 1 INTRODUCTION

Adequate access to allied health care in residential aged care (RAC), also called nursing homes, is essential to support and maximise resident well‐being and quality of life; for example, interventions delivered by physiotherapists and occupational therapists can reduce the incidence of falls,^1^ speech pathology interventions can reduce the risk of dysphagia^2^ and music therapy may improve depressive symptoms in residents with dementia.^3^ While there is no unanimous definition of allied health, it is often used to describe professions that fall outside of nursing and medical disciplines.^4^

In Australian RAC, the access to, and intensity of allied health services has been described as insufficient by professionals and consumers.^5^ Funding is a significant barrier to allied health service delivery; and over time, there has been limited commitment to change this status quo.^6^ This is not for a lack of advocacy. Rather, in a sea of services that require public funding, arguments for increased funding for allied health RAC are flawed as they lack information of funders' value, service level data and evidence.

## 2 WHAT DO WE KNOW ABOUT ALLIED HEALTH SERVICE DELIVERY IN AUSTRALIAN RAC?

To date, evidence on allied health in RAC has focused frequently on specific interventions, that is a prescribed treatment with specific frequency and duration parameters, for example, a Tai Chi program. While these studies are essential to demonstrating the benefit of allied health, they are not a good indicator of how allied health is delivered in, and the tangible benefits resulting from, day‐to‐day care in RAC.

Unfortunately, information on how much allied health is delivered in Australian RAC is scarce as data collection and reporting in the sector are not standardised.^7^ Static annual counts of the number of allied health professionals working in RAC, such as those collected at 4‐year intervals by the Department of Health,^8^ do little to capture how much face‐to‐face care is delivered in RAC. Data that present hours per resident per day of care, as recommended in the Royal Commission on Aged Care Quality and Safety, are more meaningful ways to measure service delivery, yet are currently uncommon. One 2020 cross‐sectional survey reported that 22 min of allied health were delivered per resident per day in Australian RAC.^9^ However, this sample only reflects 12% of RAC nationally and does not explore factors influencing service delivery levels such as rurality and case mix.

The lack of data on how much allied health is delivered in RAC is a critical flaw in the argument for more allied health services. From a funder's perspective, it is difficult to justify an increase when current service levels are uncertain. In the long term, national commitment to collecting allied health data will be required to address this data drought consistently. However, in the short term, allied health professionals, providers and researchers could make inroads by leveraging partnerships and using existing data, such as progress notes in electronic health records and billing data,^10^ to quantify how much allied health is delivered in RAC and by whom.

## 3 MEASURING PERFORMANCE

Without service level data it is difficult to demonstrate that more allied health, both in terms of accessibility and intensity, will yield better outcomes in Australian RAC. International evidence suggests that higher intensities of physiotherapy and occupational therapy care in RAC decrease the rate of falls with major injury, improve care quality and activities of daily living outcomes.^11^, ^12^, ^13^ Access to a dietitian also increases the residents' likelihood of meal satisfaction and their being well‐nourished.^14^

From a funder's perspective, measures of return on investment are critical information; but there are no standard outcomes by which to assess allied health services. It is likely that some outcomes in Australia's new RAC quality indicator program^15^ are relevant to allied health accessibility and intensity of care such as falls and weight loss. Data incidentally captured through the RAC quality indicator program could be used to explore the impact of allied health services on current quality indicators, such as falls and unplanned weight loss, and could inform methods by which to benchmark allied health performance.

## 4 HOW MUCH IS ENOUGH?

A logical next question funders have, when advocating for more, is how much and what mix of allied health services is adequate? Unfortunately, in the current data drought there is not enough information with which to respond. Some health systems internationally have set arbitrary targets for allied health service delivery, such as 36 min of allied health per resident per day in Ontario.^16^ In the short term, setting similar targets in Australia, as proposed to the Royal Commission,^17^ could help benchmark industry performance and, if monitored, could minimise variations in the volume of service such as those which likely occur between rural and metropolitan facilitates^18^ or to patients with certain characteristics.^19^, ^20^

Actionable targets are important to funders. However, following other health systems' targets, which also have limited evidence on impact, is not a convincing argument for funders to increase spending. An evidence‐base is urgently required to inform actionable funding recommendations that maximise care quality, resident outcomes and minimise waste. A process or outcomes‐based approach may be more appropriate than volume‐based targets to address the diverse needs of residents. Data collected on current service provision, including care plans and progress notes, and health outcomes could be used to start to explore common meaningful allied health service targets.

## 5 THE WAY FORWARD

To secure funding opportunities that will maximise residents' function and well‐being, allied health services must first present information in a language funders' value, such as cost, benefits and actionable steps. For this, data and evidence at the service level are key. Data need to focus on how allied health is provided in RAC, by whom, and its impact on resident well‐being. This could be achieved by leveraging existing electronic data collection practices and analysing routinely collected data such as progress notes, care plans, quality indicators and billing data. In the short term, results from such analyses, led by innovative RAC service provider, consumer and research partnerships, could provide a stronger evidence‐base that cannot be ignored, for future arguments to increase allied health in RAC.

## CONFLICTS OF INTEREST

No conflicts of interest declared.

## Data Availability

Not applicable.
